# A Comparative Functional Analysis of Pea Protein and Grass Carp Protein Mixture via Blending and Co-Precipitation

**DOI:** 10.3390/foods10123037

**Published:** 2021-12-07

**Authors:** Xiaohu Zhou, Chaohua Zhang, Wenhong Cao, Chunxia Zhou, Huina Zheng, Liangzhong Zhao

**Affiliations:** 1 College of Food Science and Technology, Guangdong Ocean University, Zhanjiang 524088, China; foodxiaohu@foxmail.com (X.Z.); cchunlin@163.com (W.C.); chunxia.zhou@163.com (C.Z.); zhenghn@gdou.edu.cn (H.Z.); 2 College of Food and Chemical Engineering, Shaoyang University, Shaoyang 422000, China; sys169@163.com; 3 Guangdong Provincial Key Laboratory of Aquatic Products Processing and Safety, Zhanjiang 524088, China; 4 Hunan Provincial Key Laboratory of Soybean Products Processing and Safety Control, Shaoyang 422000, China; 5 Collaborative Innovation Center of Seafood Deep Processing, Dalian Polytechnic University, Dalian 116034, China

**Keywords:** pea, protein co-precipitates, protein blends, surface hydrophobicity, emulsifying properties

## Abstract

Currently, the application of protein mixture derived from plants and animals is of great interest to the food industry. However, the synergistic effects of isolated protein blends (BL) are not well established. Herein, the development of a more effective method (co-precipitation) for the production of protein mixtures from pea and grass carp is reported. Pea protein isolate (PPI), grass carp protein isolate (CPI), and pea–carp protein co-precipitates (Co) were prepared via isoelectric solubilization/precipitation using peas and grass carp as raw materials. Meanwhile, the BL was obtained by blending PPI with CPI. In addition, the subunit composition and functional properties of Co and BL were investigated. The results show that the ratios of vicilin to legumin α + β and the soluble aggregates of Co were 2.82- and 1.69-fold higher than that of BL. The surface hydrophobicity of Co was less than that of BL, PPI, and CPI (*p* < 0.05). The solubility of Co was greater than that of BL, PPI, and CPI (*p* < 0.05), and the foaming activity was higher than that of BL and CPI (*p* < 0.05) but slightly lower than that of PPI. In addition, based on the emulsifying activity index, particle size, microstructure, and viscosity, Co had better emulsifying properties than BL, PPI, and CPI. The study not only confirmed that co-precipitation was more effective than blending for the preparation of mixed protein using PPI and CPI but also provided a standard of reference for obtaining a mixture of plant and animal proteins.

## 1. Introduction

The increasing demand for high-quality food-grade proteins exhibiting acceptable nutritional and functional properties represents a challenge. Animal proteins are considered to be high-quality proteins but are expensive to produce, with an energy input to protein ratio of 14:1 [1]. Plant proteins may be an economical option to meet the increasing scarcity of protein resources [2]. However, the exclusive use of plant-derived protein is not optimal from a nutritional perspective, as essential amino acid requirements are not met. Nonetheless, mixing plant protein with animal protein can sustain the nutritional requirements of humans. Increasingly, the combination of plant and animal proteins is found to result in a synergistic benefit, which makes the design of foods with both plant and animal proteins a research hotspot [3].

**Figure 1:**
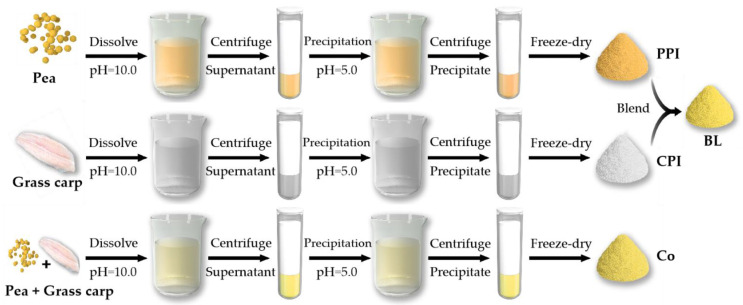
Schematic diagram of the preparation of the pea protein isolate (PPI), grass carp protein isolate (CPI), protein blends (BL), and protein co-precipitates (Co).

Mixed proteins can be classified into two forms, protein blends (BL) and protein co-precipitates (Co) [4]. The differences in the preparation methods used to generate Co and BL are presented in Figure 1. BL refers to the blending of isolated proteins. Mixtures of pea protein isolate (PPI) and cod protein isolate as well as the combination of soy protein isolate and whey protein isolate exhibit synergistic emulsifying effects; however, when the proportion of plant protein replacing animal protein is increased, the synergistic effect is weakened, indicating that BL application has certain limitations [5,6]. Some studies suggest that the emulsifying gelation of directly mixed plant and animal proteins has an obvious antagonistic effect [7,8]. Concerning the preparation of Co, heterologous proteins are processed via isoelectric solubilization/precipitation (ISP) [9]. Driven by pH, proteins from different sources are simultaneously dissolved and precipitated to promote interaction between heteroproteins; generate additional disulfide bonds; change the subunit composition [10]; and alter the surface charge, solubility, and surface hydrophobicity of the proteins, thus effectively improving their functional properties [11]. Studies have found that legume–rapeseed Co has better nutritional and functional properties than single protein [12], and soy–whey protein Co has high water-holding and gel formation capacity [13]. Previously, we investigated the functional properties of soy–tilapia Co and found that the solubility and emulsifying properties of the Co were better than those of the single protein [14,15,16]. However, there are few comparative studies of BL and Co.

Peas (*Pisum sativum* L.) are one of the main legumes grown worldwide, with an annual global output of approximately 14.2 million tons [17]. Peas containing 20–30% protein are rich in lysine but deficient in methionine. Moreover, compared with soybean, pea protein is not associated with a risk of sensitization or transgenic origin. PPI has better emulsifying properties than soybean protein isolate under most pH values and protein concentrations [18]. Grass carp (*Ctenopharyngodon idellus*) is one of the four major carps in China; it is widely consumed in Asian countries and is rich in methionine. Grass carp protein isolate (CPI) can inhibit the flocculation of oil droplets and oxidization of lipids. Therefore, CPI is a potential emulsifier. However, it has poor stability and must be modified or mixed with other proteins for various industrial applications [19,20]. In order to conduct a broader study, pea and grass carp mixed proteins were selected, based on the cost of raw material and the functional properties of the proteins.

The properties of proteins are easily influenced by environmental conditions. pH is the primary factor affecting the properties of proteins, and it regulates electrostatic interactions. It drives the adsorption, reorientation, alignment, and formation of viscoelastic films on the interface, thus changing the stability of the interface [21]. This study evaluated the effects of blending and co-precipitation on the functional properties of pea and grass carp mixed protein by analyzing the solubility, foaming, and emulsifying properties of BL and Co at different pH values. The goal was to provide a basis for the subsequent development of products containing plant and animal proteins.

## 2. Materials and Methods

### 2.1. Materials

Split peas were purchased from Foshan Jinnuoyi Processing Factory of Agricultural Products (Foshan, China); the protein content was 21.12% (6.25 of N, wet weight). Grass carp were purchased from the local fish market in Zhanjiang City, Guangdong Province. The gills, heads, tails, bones, and blood were removed, leaving only the white muscles on the back, which were retained and frozen at a low temperature. The fish muscles were brought back to the laboratory and stored at −20 °C; the protein content was 18.27% (6.25 of N, wet weight). Electrophoresis reagents were purchased from Beyotime Biotechnology Co., Ltd. (Shanghai, China). The ANS fluorescence probe was purchased from Shanghai Aladdin Biochemical Technology Co., Ltd. (Shanghai, China). Other reagents were of analytical grade and were ordered from the Guangzhou Chemical Reagent Factory (Guangzhou, China).

### 2.2. Preparation of PPI, CPI, Co, and BL

Protein samples were prepared using the ISP method [14]. Peas were powdered and sieved through a 100-mesh sieve (0.15 mm). The pea powder was mixed with deionized water at 4 °C at a ratio of 1:9 (*w/v*). The pH was adjusted to 10.0 with 1 M NaOH and dissolved for 30 min, followed by centrifugation at 10,000× *g* and 4 °C for 20 min. The precipitate was removed and 1 M HCl was slowly added to the supernatant. The mixture was fully stirred, and the pH was adjusted to 5.0. At this time, the precipitation was washed with deionized water, and the pH value was adjusted to 7.0, followed by dialysis. PPI was obtained after freeze-drying for 48 h. The fish muscles were defrosted and chopped, and CPI was prepared according to the steps indicated above for PPI. The pea powder was mixed with fish muscle, and the Co was prepared following the steps described above. BL was obtained by directly mixing the powdered PPI and CPI. To ensure similar comparison, the protein ratio of pea and grass carp in BL and Co was maintained at 1:1 (W:W) in this study.

### 2.3. Sodium Dodecyl Sulfate Polyacrylamide Gel Electrophoresis (SDS-PAGE)

Similar concentrations of BL and Co solutions were prepared. Reducing and non-reducing SDS-PAGE gels were used with 5% spacer gel (upper gel) and 12% separation gel (lower gel) on a vertical gel electrophoresis apparatus at 15 mA. The difference between reducing and non-reducing electrophoresis depends on whether or not the reducing agent dithiothreitol (DTT) is added to the loading buffer. Standard protein markers (16–270 kDa) were used for qualitative analysis of the protein bands in the samples. The protein bands were dyed with Coomassie brilliant blue (CBB) R-250. Later, 40% methyl alcohol and 40% acetic acid were used for decoloration. The relative strength of the dyed protein bands was measured using ImageLab (Bio-Rad Laboratories, Hercules, CA, USA).

### 2.4. Solubility

According to Kristinsson’s method [22], a 0.5% protein solution was prepared. The pH was adjusted to 3.0, 5.0, 7.0, 9.0, and 11.0 and then stirred for 30 min after centrifuging at 10,000× *g* for 20 min. Bovine serum albumin (BSA) was used as the standard and the protein content in the supernatant was measured using the Lowry method at 750 nm. The solubility was calculated as follows:

Solubility (%) = *C*/*C*_0_ × 100%, (1)

where *C* is the protein content in the sample after centrifugation (mg) and *C*_0_ denotes the protein content in the sample before centrifugation (mg).

### 2.5. Surface Hydrophobicity (H_0_)

The protein surface hydrophobicity was measured using the ANS fluorescence probe as described by Haskard and Li-Chan [23]. A 50 mL aliquot of 0.5% protein solution was added to 10 mmol/L phosphate buffer with pH values of 3.0, 5.0, 7.0, 9.0, and 11.0. Then, the mixtures were stirred for 1 h at room temperature, followed by centrifugation for 30 min at the rate 7500 rpm. The protein concentrations of the supernatant were measured using the Lowry method. After dilution with the same phosphate buffer solution (PBS) (concentration ranging from 0.005 to 0.5 mol/mL), different concentrations of sample solutions were collected (4 mL), to which 40 μL of 8 mmol/L ANS solution (10 mmol/L, prepared with pH 7.0 PBS) was added. After oscillation, the mixtures were held static for 3 min, and the fluorescence intensity of the samples was tested using a G9800A fluorescence spectrophotometer (Cary Eclipse, Agilent, Santa Clara, CA, USA). In this test, the excitation and emission wavelengths were λex = 370 nm and λem = 490 nm, respectively, and the crack was 5 nm. Graphs of fluorescence intensity relative to the protein concentration were plotted. The slope of the initial section is equal to the surface hydrophobicity (*H*_0_) of the protein molecules.

### 2.6. Foaming Properties and Foaming Stability

With reference to the method of Fekria et al. [24], 1.0% protein solutions were prepared, and the pH was adjusted to 3.0, 5.0, 7.0, 9.0, and 11.0. The protein solutions were stirred at room temperature for 30 min, and specific volumes of the protein solution (V_0_) were collected and emulsified with the homogeneous shear machine at a rate of 10,000 r/min. The protein solutions were transferred immediately to the tube to measure the foam volume (*V*_1_). After resting for 30 min, the foam volume (*V*_30_) was again measured. The foaming capacity (FC) and foaming stability (FS) were as follows:

(2) $$\mathrm{FC}\;=\frac{V_{1}-V_{0}}{V_{0}}\times 100\%\;,$$

(3) $$\mathrm{FS}\;=\frac{V_{30}}{V_{1}}\times 100\%\;,$$

### 2.7. Emulsion Preparation

A 1.0% protein solution was prepared, and the pH was adjusted to 3.0, 5.0, 7.0, 9.0, and 11.0. The solutions were stirred and dissolved for 30 min and then mixed with soybean oil at a ratio of 9:1 (*v/v*). The mixed proteins were blended with a T18 homogeneous shear machine (ULTRA-TURRAX, IKA, GER) for 1 min at a rate of 9000 rpm, followed by additional emulsification for 1 min at a rate of 15,000 rpm. The emulsions were stored under 4 °C.

### 2.8. Emulsifying Activity Index (EAI) and Emulsifying Stability Index (ESI)

The EAI and ESI of the proteins were measured via turbidimetry [25]. A 0.5% protein solution was prepared, and the pH was adjusted to 3.0, 5.0, 7.0, 9.0, and 11.0. The solutions were stirred and dissolved for 30 min. The protein solutions (6 mL) were mixed with soybean oil (2 mL) and emulsified with the homogeneous shear machine for 1 min (12,000 rpm). Subsequently, 20 μL of each emulsion was collected from the bottom container and dispersed in 4 mL of 0.1% SDS. The absorbance (*A*_0_) was measured at a wavelength of 500 nm. After 10 min, 20 μL of the emulsion was again collected from the bottom container and was dispersed in 4 mL of 0.1% SDS. The absorbance (*A*_10_) was tested at a wavelength of 500 nm. The blank control was 0.1% SDS. The EAI and ESI were calculated as follows:

(4) $$\mathrm{EAI}\;=\frac{2\times 2.303\times A_{0}\times D}{C\times \varphi \times 10^{4}}(m^{2}/g)$$

(5) $$\mathrm{ESI}\;=\frac{A_{0}}{A_{0}-A_{10}}\times \Delta t\;(\min)$$

where *D* is the dilution ratio (200), *C* denotes the protein concentration (5 mg/mL), φ represents the volume fraction of oil in the emulsion (0.25), L refers to the optical cuvette path length (1 cm), and Δt indicates the measurement interval (10 min).

### 2.9. Particle Size and Zeta Potential

The particle size distribution of the emulsion, volume-weighted average particle size (*d*_4,3_), surface-area-weighted average particle size (*d*_3,2_), and zeta potential under different pH values were measured with a Malvern Zetasizer Nano ZS (Malvern Panalytical, Malvern, UK). The relative refractive index of the emulsion was 1.107, while the refractive indices of soybean oil and water were 1.472 and 1.330, respectively. The average particle size distribution of the emulsification was characterized by *d*_4,3_. Further, *d*_4,3_ and *d*_3,2_ values were measured using deionized water as the dispersing agent. The values of *d′*_4,3_ and *d′*_3,2_ were measured using 1.0% SDS as the dispersing agent.

### 2.10. Interface Protein Absorption (AP) and Interface Protein Content (Γ)

AP and Γ were measured according to the methods of Liang and Tang [26]. The fresh emulsion (1 mL) was centrifuged for 30 min at a rate of 10,000 r/min, resulting in separation into an upper layer of greasy emulsion and a lower layer of protein supernatant. The upper layer was eliminated carefully, and the lower supernatant was collected with an injector and filtered through a 0.22 μm needle-shaped filter. BSA was used as the standard protein, and the protein concentration of the lower supernatant was tested with the Lowry method. The AP and Γ were calculated as follows:

(6) $$\mathrm{AP}\;=\frac{C_{0}-C_{s}}{C_{0}}\times 100\%\;,$$

(7) $$\Gamma \;=C_{0}-C_{s}\frac{1-\varphi }{6\varphi }{d^{\prime }}_{3,2\;},$$

where *C*_0_ is the protein concentration in the protein solution used to prepare the emulsion (10 mg/mL); *C_s_* denotes the protein concentration of the lower supernatant layer after centrifuging the emulsion (mg/mL); Γ represents the interface protein content (mg/m^2^); φ stands for the oil volume fraction (0.1), and *d′*_3,2_ refers to the surface area average particle size of the emulsion, which was measured using 1.0% SDS as the dispersing agent (μm).

### 2.11. Confocal Laser Scanning Microscopy

The microstructure of the emulsion was analyzed via confocal laser scanning microscopy (CLSM; FV1200, Olympus, Japan) according to the method of Liu [27]. The emulsion (20 μL) was collected and added to a centrifuge. Simultaneously, 10 μL of 0.1% (*w/v*) Nile red methanol solution and 10 μL 1.0% (*w/v*) Nile blue aqueous solution were added; the reaction was performed in the dark for 15 min. The mixture (10 μL) was collected to produce films and observed with a 100× oil immersion lens. The resolution of the scanning images was 1024 × 1024. Proteins were stained with Nile blue, and laser excitation was performed at an excitation wavelength of 633 nm and a detection wavelength of 655–755 nm. Oil phase dyeing was performed with Nile red, followed by laser excitation at an excitation wavelength of 559 nm and a detection wavelength of 570–625 nm.

### 2.12. Rheological Properties

The rheological properties of the emulsion were measured with a rheometer (Discovery DHR-2 Dynamic Shear Rheometer, TA, Milford, MA, USA). A 40 mm plate was used to test the apparent viscosity of the emulsion within the shear rate range of 0.1–200 s^−1^.

### 2.13. Statistical Analysis

All experimental measurements were performed at least three times. SPSS 25.0 was used for all statistical analyses. Statistical differences between two groups were determined with one-way analysis of variance (ANOVA) and Duncan’s multiple range test (*p* < 0.05). The results are expressed as the mean ± standard deviation (SD).

## 3. Results and Discussion

### 3.1. Protein Composition Analysis

**Figure 2:**
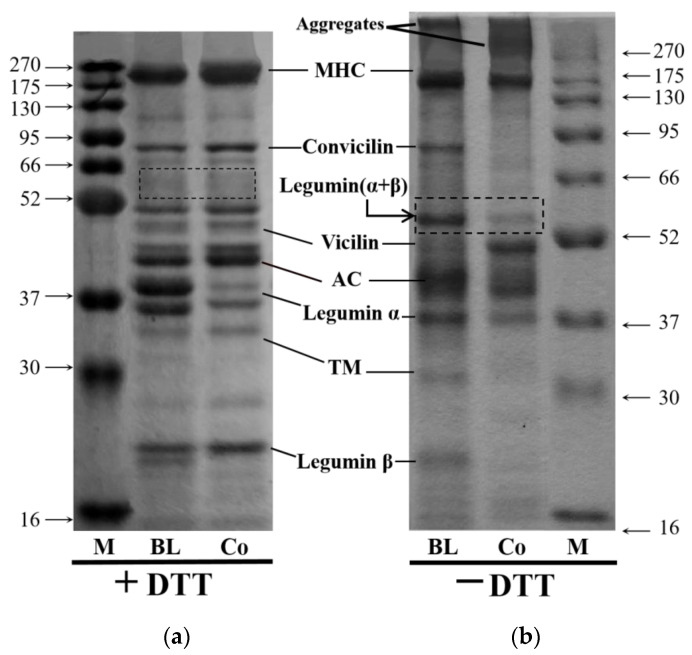
Reducing (**a**) and non-reducing (**b**) electrophoretograms of protein blends (BL) and protein co-precipitates (Co).

The protein compositions of BL and Co were analyzed via reducing and non-reducing SDS-PAGE. As can be seen in Figure 2 and Table 1, both BL and Co contained myosin heavy chain (MHC, ~200 kDa), convicilin (~70 kDa), legumin (~62 kDa), vicilin (~50 kDa), actins (AC, ~42 kDa), legumin α (~38 kDa), tropomyosin (TM, ~34 kDa), legumin β (~22 kDa), and aggregates consistent with the literature [28,29]. To sum up, both BL and Co effectively combined pea and CPI, and thus, both are suitable for further testing.

Reducing SDS-PAGE reflects the specific subunit composition of proteins. BL and Co were both composed of pea and carp (1:1). However, the MHC, legumin α, and legumin β protein bands differed significantly in reducing SDS-PAGE, indicating that Co and BL showed different degrees of crosslinking or degeneration with subunits of CPI. Similar studies have suggested that such differences might be attributed to different electrostatic and hydrophobic interactions and the effects of disulfide bond in proteins during preparation of Co by the ISP method [10]. Co contains a higher number of MHCs, indicating that its functional properties might have some advantages [30]. The reduced MHC retention of Co in non-reducing electrophoresis indicates that some of the MHC components of Co entered soluble aggregates. Legumin is composed of six pairs of subunits and each pair contains an α-polypeptide (acid subunit) and a β-polypeptide chain (alkaline subunit) linked via a disulfide bond. The disulfide bond can only be opened under reducing conditions, thus dissociating the protein subunits [31]. SDS can decompose hexamer legumin to 60 kDa dimer legumin α + β under heating conditions. However, DTT destroys the non-covalent bonds of the legumin hexamer as well as the disulfide bonds between the α- and β-polypeptide chains. Hence, no legumin α + β band is detected at 60 kDa in the reducing SDS-PAGE. In contrast, the disulfide bond between the α- and β-polypeptide chains remains intact in the absence of DTT [32]. Therefore, legumin α + β bands appeared at the position of 60 kDa following non-reducing electrophoresis. The optical density of Co was significantly less than that of BL, indicating that legumin α + β components of Co also partially entered the aggregate.

Non-reducing electrophoresis cannot disrupt the disulfide bonds. Therefore, it reflects the original composition of the various proteins in a mixed protein. Compared with proteins separated via reducing SDS-PAGE, many soluble aggregates were blocked at the top of the separated gel and the concentration of Co (19.62%) was 1.69-fold higher than that of BL (11.61%) (Table 1), indicating the presence of additional bonds in Co. Simultaneously, as mentioned above, some of pea and grass carp proteins entered the aggregates, indicating that co-precipitation leads to the formation of disulfide bonds between pea protein and grass carp protein, resulting in altered subunit composition. Current studies indicate that the formation of disulfide bonds between globulin molecules enhances the flexibility of the protein structure and improves its foaming and emulsifying capacity as well as its stability. When proteins are adsorbed onto the O/W interface, the stability of the emulsion is improved [33]. Compared with legumin α + β, vicilin is more flexible and exhibits better interfacial activity [34]. Many studies have confirmed that the vicilin/legumin α + β ratio is positively related to a protein’s functional properties, including solubility, and foaming and emulsifying properties [35,36]. Thus, the ratio of vicilin to legumin α + β is very important. As shown in Table 1, the proportion of Co (203.99%) is 2.82-fold larger than that of BL (72.36%). Therefore, the analysis of protein composition indicates that the functional properties of Co might be better than those of BL.

### 3.2. Solubility and Surface Hydrophobicity (H_0_)

**Figure 3:**
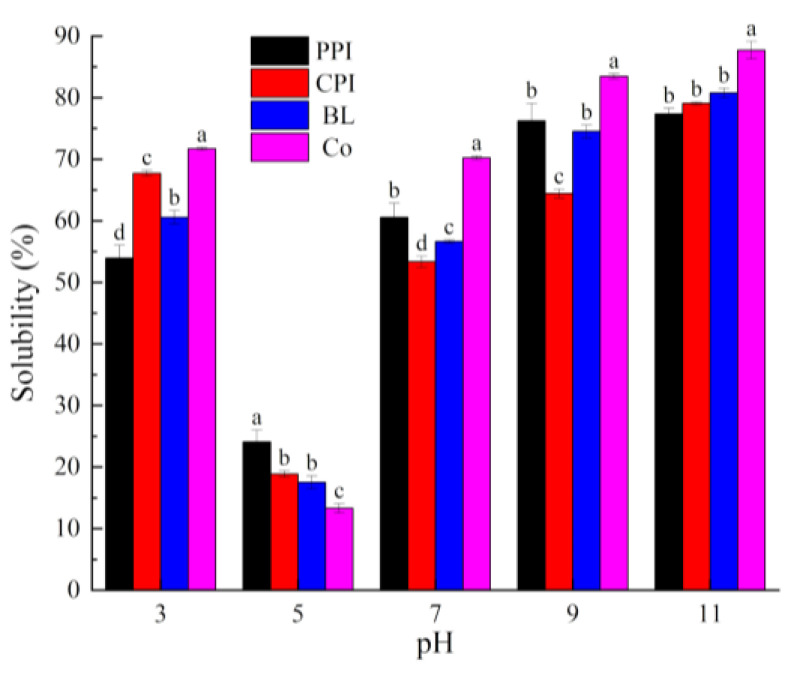
Solubility of proteins under different pH values. Different letters under the same pH value indicate significant differences (*p* < 0.05).

Solubility is a key functional characteristic that can be used to evaluate the potential use of protein in food and determine its foaming, emulsification, and gel formation ability [37]. As can be seen in Figure 3, the solubility of Co, BL, PPI, and CPI was significantly pH dependent, with solubility exhibiting a U-shaped curve. The minimum solubility was observed at pH 5.0 because the isoelectric point (PI) of globulin is 4.5 and the isoelectric point of myofibrillar protein is 5.3, which is consistent with other studies of PPI [38], CPI [39], and soy–fish mixed protein [14]. When the pH deviates from the isoelectric point, solubility significantly increases because an increase in net charges on the protein surface reinforces the intermolecular electrostatic repulsive force, which promotes ion–dipole interaction between proteins and water molecules [40].

**Figure 4:**
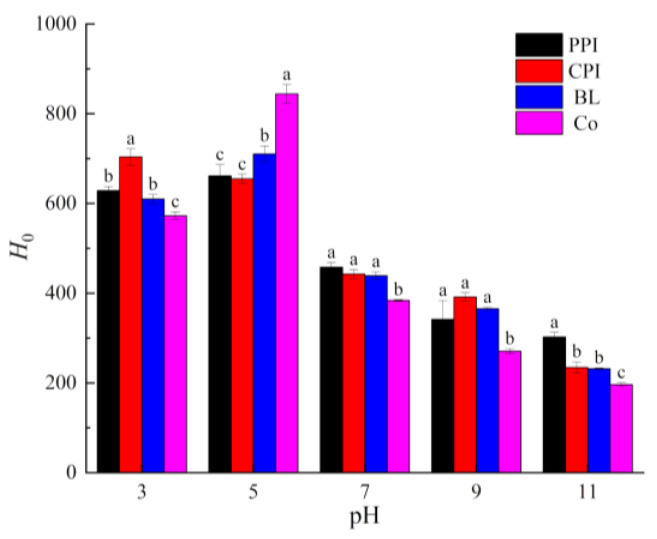
Surface hydrophobicity (*H*_0_) of proteins under different pH values. Different letters under the same pH value indicate significant differences (*p* < 0.05).

ANS is a fluorescence probe that is sensitive to external polar groups and interacts with hydrophobic groups of protein molecules to generate fluorescence spectra, which can be used to characterize the exposure of hydrophobic groups in proteins. As shown in Figure 4 under different conditions, *H*_0_ varied inversely with solubility. Thus, a higher solubility was associated with fewer hydrophobic groups on the protein molecular surface [41].

Comprehensive analysis of the solubility and surface hydrophobicity of the mixed proteins indicated that the solubility of Co was the highest under all pH values except at pH 5.0 (*p* < 0.05), which is similar to the result of our previous study of soy–tilapia Co [14]. Co prepared by ISP was precipitated at pH 5.0. Under this circumstance, pea and CPI molecules carry opposite charges and the intermolecular electrostatic interaction is strengthened to generate soluble Co-aggregates of proteins, triggering the reconstruction of hydrophobic and disulfide bonds [42]. Moreover, some subunits, such as vicilin, will be concentrated “selectively”, thus changing the protein properties [43]. BL is formed by mixing PPI and CPI powders, and the molecular interaction is not as strong as that of Co. The molecular interaction of BL involved PPI and CPI at pH 3.0, 7.0, and 9.0, but the solubility (80.77%) at pH 11.0 was a little higher than that of PPI (77.36%) and CPI (79.07%). Under strong alkaline conditions, protein molecules degenerate, and hydrophobic groups are exposed. Protein molecules cluster with each other via hydrophobic bonds, inducing hydrophobic nonlinear superposition [44]. As a result, the *H*_0_ (231.53) of BL was lower than that of PPI (302.36) and CPI (234.41). Due to this hydrophobic synergistic effect, the solubility of BL was higher than that of PPI and CPI. Thus, Co has a better synergistic effect on solubility than BL.

### 3.3. Foaming Properties

**Figure 5:**
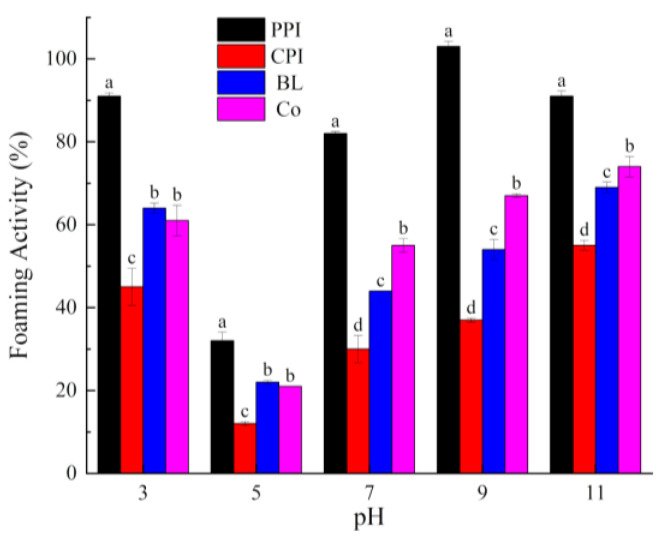
Foaming capacity of proteins under different pH values. Different letters under the same pH value indicate significant differences (*p* < 0.05).

**Figure 6:**
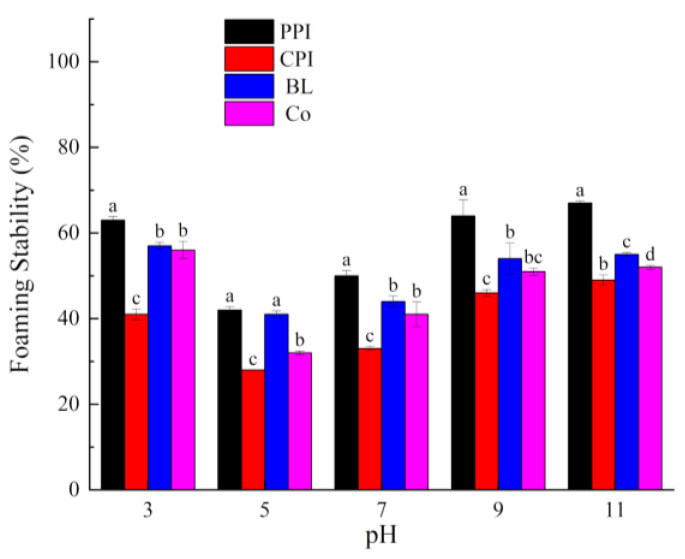
Foaming stability of proteins under different pH values. Different letters under the same pH value indicate significant differences (*p* < 0.05).

Foaming plays a critical role in the texture and structure of foods, such as ice cream, cakes, breads, and meringue. Currently, foaming is achieved by replacing a surfactant with protein. Foaming capacity (FC) and foaming stability (FS) are the most common indices used to describe the foaming properties of protein [45]. The effects of pH on the FC and FS of the proteins are shown in Figure 5 and Figure 6. Overall, FC and FS exhibited a U-shaped variation, similar to those observed for solubility. The foaming properties were the weakest at pH 5.0, which is consistent with other studies [46]. When the pH deviated from the PI, the solubility of the protein was improved and the degree of dispersion of the protein molecules in water was increased. Under these circumstances, the surface charges on the protein molecules are increased and the protein structures are opened to expose the hydrophobic amino acid side chains. Hence, the proteins quickly locate at the air–water interface, and the surface tension is decreased, thus improving the FC and FS of the proteins.

Zhao et al. argued that the FC of PPI was similar to that of soy protein isolate, but the FS was slightly higher. PPI is a plant foaming agent with potential application in various food preparations [47]. In this study, the FC and FS of PPI were the highest under all pH values (*p <* 0.05). The maximum value of FC was 103 ± 1.25 at pH 9.0, which is related to the small molecular weights of PPI, small volume, and weaker intermolecular interaction [48]. CPI exhibited the opposite trend. The molecular weights of fibrillin are relatively high and present a linear structure, thus causing significant steric hindrance. The air–water interfacial absorption capacity of fibrillin is significantly lower than that of globulin, and the compactness of the viscous layer is insufficient. Therefore, the FC and FS of fibrillin are relatively low [49]. The foaming performance of the mixed protein was better than that of CPI, but still inferior to that of PPI at all pH values, i.e., there was no synergistic effect, similar to solubility. The FC of Co was lower than that of BL at pH 3.0 and 5.0, and the FS of Co was lower than that of BL under all pH values. This might be because Co contains additional macromolecular soluble aggregates, making it difficult to form a dense viscous layer on the water–air interface. In brief, the addition of PPI is a potential strategy to improve the foaming properties of CPI.

### 3.4. Emulsifying Properties

#### 3.4.1. EAI and ESI

**Figure 7:**
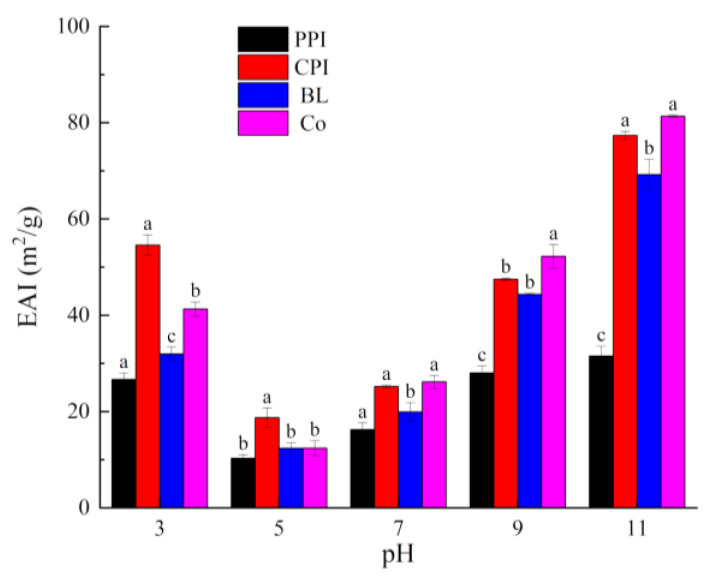
Emulsifying activity index of proteins under different pH values. Different letters under the same pH value indicate significant differences (*p* < 0.05).

**Figure 8:**
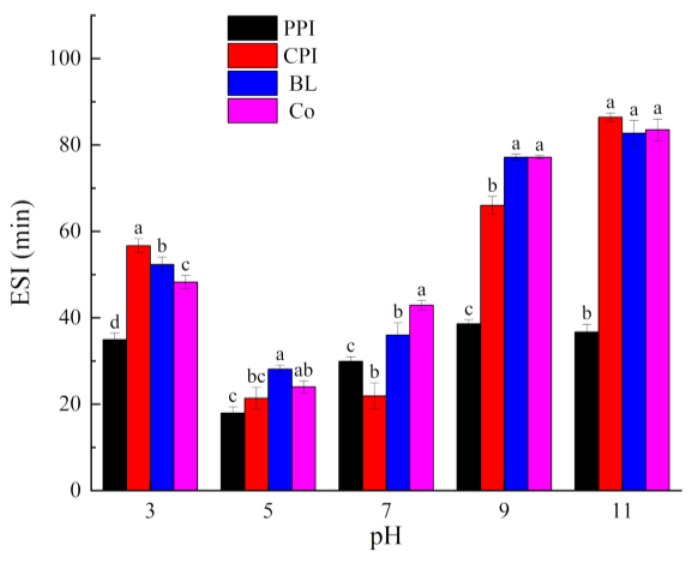
Emulsifying stability index of proteins under different pH values. Different letters under the same pH value indicate significant differences (*p* < 0.05).

EAI refers to the oil–water interface area for stabilization of the unit mass of the emulsifier; it reflects the resistance of a protein to emulsion stratification caused by flocculation and aggregation. Thus, the EAI can be used to evaluate the emulsifying properties of proteins. ESI refers to the ability of proteins to remain in a stable state within a certain period, without phase layering or separation [25]. EAI and ESI are key indices that reflect the functional properties of proteins. As shown in Figure 7 and Figure 8, the EAI and ESI of all proteins first decreased and then increased under all pH values. This phenomenon is similar to the trend of the solubility and foaming properties, but contrary to the *H*_0_ value. The emulsifying properties and emulsifying stability near PI were the lowest because, at PI, the protein surface carries few charges and lacks electrostatic repulsion. Accordingly, proteins aggregate, and flocculation occurs [50]. Increasing pH can lead to the degeneration of proteins. The protein molecular conformation in the “melting state” is soft and relaxed. The residual groups of the exposed hydrophobic amino acids point to the oil phase, while the hydrophilic groups point to the water phase, thus enhancing the interaction with oil phases [51]. As a result, the emulsifying properties are enhanced. It can be seen that the pH changes the ability of proteins to stabilize oil droplets via expansion and adsorption on interfaces by regulating electrostatic interactions and *H*_0_ for stability.

The ESI values of BL and Co did not exhibit any clear pattern. At pH 3.0, the ESI of BL was higher than that of Co (*p* < 0.05). At pH 7.0, the ESI of BL was lower than that of Co (*p* < 0.05). There were no significant differences at pH 5.0, 9.0, and 11.0 (*p* > 0.05). However, testing the particle size of the emulsions and analysis under CLSM revealed that Co stabilized smaller oil droplets (discussed later), indicating improved stability. The testing time of ESI was only 10 min, which was not sufficient to complete the maturation process and allow adsorption of the protein on the interface.

#### 3.4.2. Particle Size and Zeta-Potential Analysis

**Figure 9:**
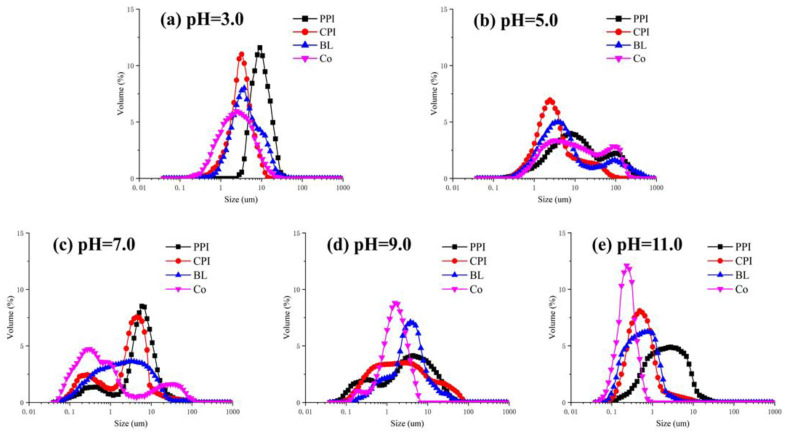
Droplet size distribution of proteins under different pH values. (**a**) pH = 3.0; (**b**) pH = 5.0; (**c**) pH = 7.0; (**d**) pH = 9.0; (**e**) pH = 11.0.

**Figure 10:**
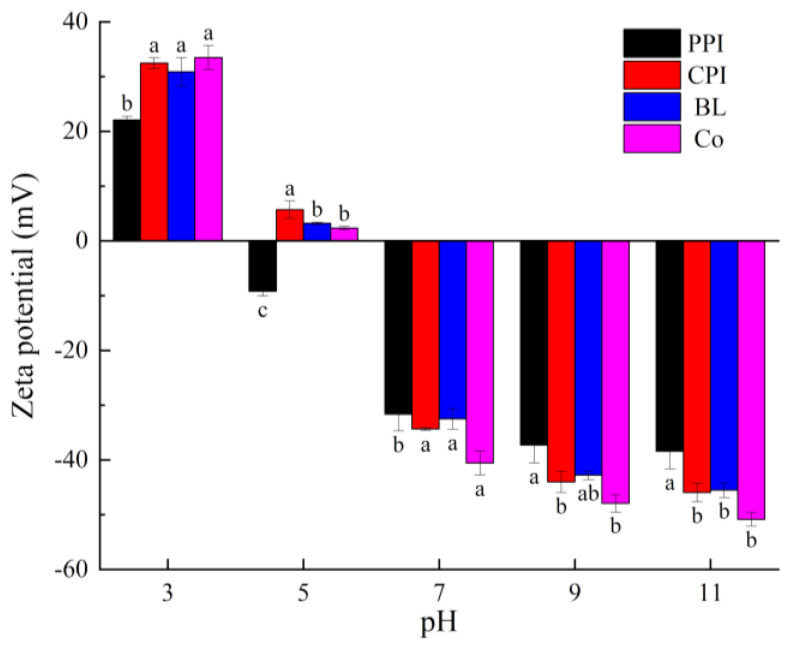
Zeta potential of proteins under different pH values. Different letters under the same pH value indicate significant differences (*p* < 0.05).

Particle size is an important index used to measure the stability of an emulsion [52]. The particle size distributions of protein emulsions were evaluated using the volume-weighted mean diameter (*d*_4,3_) (Figure 9), supplemented with the surface-area-weighted mean diameter (*d*_3,2_), and *d′*_43_ and *d′*_32_ values of the emulsion with 1% SDS (Table 2). The effect of the pH value on the particle size was analyzed. The results indicated that the *d*_4,3_ values of the emulsions at pH 3.0 and 5.0 were relatively high (3.29 ± 0.27 μm and 22.79 ± 2.75 μm for Co) because the electrostatic repulsion among proteins is insufficient to overcome attractions (Figure 10), thus resulting in particle clusters and increasing the particle size. According to Laplace theory, the droplet size of an emulsion decreases with a reduction in two-phase interface tension under constant homogeneity [53]. The *d*_4,3_ values of the emulsions at pH 9.0 and 11.0 were significantly lower than at other pH values (0.94 ± 0.05 μm and 0.54 ± 0.01 μm for Co), which is consistent with a previous study reporting a significant decrease in the *d*_4,3_ of soy protein isolate under alkaline pH (pH 9.0–12.0) [54]. Small particle size is conducive to the formation of a stable emulsion [55]. Therefore, the most stable emulsion systems were achieved at pH 11.0. Moreover, as shown in Figure 9 and Figure 10, all proteins exhibited single-peak particle size distributions at pH 3.0 and 11.0, which is related to the high net charges. At pH 5.0 and 7.0, relatively large particle size peaks (10–100 μm) were observed. Under these conditions, oil droplets develop flocculence, which is attributed to bridging protein aggregates between the droplets. Alternatively, it is difficult to achieve ripening and saturation on the oil–water interface due to the low protein–oil ratio, thus leading to bridging flocculation [56]. The particle size of CPI under all pH values was smaller than that of PPI, which is consistent with the results of EAI, because the molecular flexibility of myofibrillar protein (MP) is higher than that of globulin and, thus, leads to directional expansion on the oil–water interface. Therefore, CPI can stabilize smaller oil droplets. The particle size of Co was smaller than that of BL under all pH values, except for pH 5.0 (*p* < 0.05), which demonstrates the higher emulsifying capacity of Co. The trends in *d*_3,2_, *d*′_4,3_, and *d′*_3,2_ variation were similar to that of *d*_4,3_.

Zeta potential is an index of particle repulsion or attraction intensity. The higher the absolute zeta-potential value, the higher the stability of an emulsion [57]. The zeta potentials of droplets under different pH values were evaluated (Figure 10). For all samples, the zeta potential changed from positive to negative when the pH increased from 3.0 to 11.0. This trend can be attributed to the PI values of PPI and CPI, which were 4.5 and 5.2, respectively. At pH 5.0, the zeta potentials of the emulsions were the lowest (2.32 ± 0.31 mV for Co), and the net charges were the lowest, which is related to the negatively charged aggregates on the interfaces. The zeta potentials of the emulsions increased significantly when the pH increased from 7.0 to 11.0 because when the pH is higher than the PI of a protein, electrostatic repulsion increases, and flocculation of droplets is inhibited. Consistent with the particle size results, the zeta potential of CPI was higher than that of PPI (*p <* 0.05). The zeta potential of Co was higher than that of BL under all pH values, except at pH 5.0, which is attributed to the better dispersing capacity of Co and the higher sensitivity of charging properties to pH. This is related to AP and Γ.

#### 3.4.3. Interface Protein Adsorption (AP) and Interface Protein Content (Γ)

AP refers to the percentage ratio between the protein content adsorbed on the oil droplet surface and the protein content in the continuous phase. Γ refers to the protein content on the unit area of a droplet [43]. The AP (%) and Γ values of PPI, CPI, BL, and Co under different pH values are listed in Table 3. At pH 5.0, the AP (%) of all four protein emulsions was the lowest (2.96–11.42%), indicating the presence of abundant protein isolates in the emulsions. The protein solubility decreased due to the electrostatic shielding effect at pH 5.0. When the pH deviated from the PI, the AP increased gradually, which is consistent with the study of Liang and Tang [43]. The Γ value of an emulsion reflects the thickness of the stable oil–droplet interface membrane. Although it is not directly correlated with the particle size of emulsion droplets or AP, it is still influenced by solubility. The overall trends of variation were also similar to those of AP. Nevertheless, it is interesting that the AP of BL at pH 5.0 was higher than at pH 7.0, and the AP of Co at pH 5.0 was higher than at pH 3.0 and 7.0. This indicates that, during emulsification via high-speed shearing, insoluble proteins can also adsorb onto the interface and an emulsion with a similar stable Pickering effect is formed [58]. The results indicated that the AP% and Γ of CPI were higher than those of PPI, and the AP% and Γ of Co were higher than those of BL. These results further demonstrate that surface hydrophobicity is a major and decisive factor influencing the emulsifying properties of a protein under high solubility.

#### 3.4.4. Confocal Laser Scanning Microscopy (CLSM)

**Figure 11:**
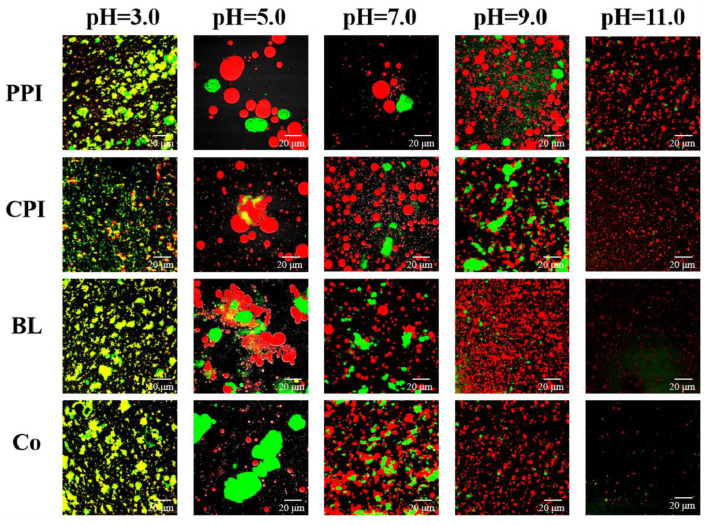
Micromorphology of protein emulsions under different pH values.

CLSM is often used to analyze the microstructure of an emulsion; it reflects the original particle size distribution, dispersion, and stability of emulsion particles. The red and green zones represent oil (Ar laser 559 nm) and protein (He/Ne laser 633 nm), respectively. The microstructures of the protein emulsions under different pH values are shown in Figure 11. The overall results were consistent with the observed particle size results. Almost all oil droplets were spherical and wrapped in an outer protein layer. At pH 5.0, all four protein emulsions showed serious flocculation. The emulsion particles were large and irregular and were mostly distributed in clusters. Although the oil droplets stabilized by Co were small, evident protein aggregation was observed. A pH value approaching PI is the main cause for the bridging flocculation of oil droplets and proteins [6]. When the pH was far from the PI, electrostatic repulsion increased with the increase in the solubility of the protein, while the particle size of the emulsion droplets decreased rapidly. Similarly, the emulsion particle sizes stabilized by BL and Co were smaller and distributed more uniformly under the same pH (e.g., pH 9.0 and 11.0), which is consistent with the results obtained with soy–whey mixed protein at pH 2.0–11.0 [6]. The variation trend of Co was more obvious. CLSM directly validated the EAI/ESI, AP/Γ, and the results of particle size distribution, indicating that Co has a better emulsifying effect than BL.

#### 3.4.5. Rheological Properties

**Figure 12:**
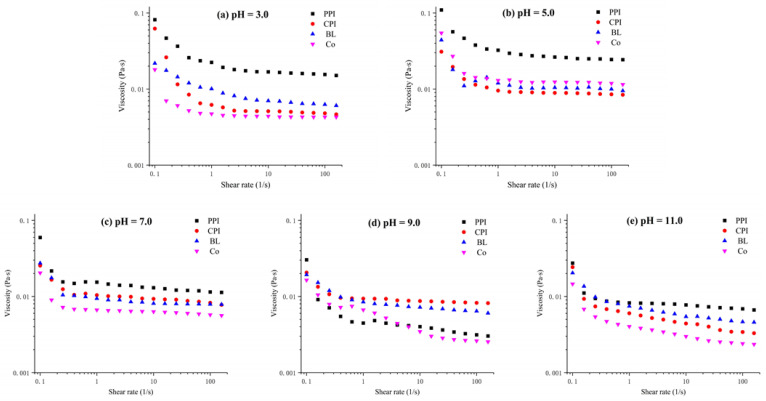
Apparent viscosity of protein emulsions under different pH values. (**a**) pH = 3.0; (**b**) pH = 5.0; (**c**) pH = 7.0; (**d**) pH = 9.0; (**e**) pH = 11.0.

The processing and storage stability of an emulsion can be predicted based on the apparent rheological properties of the emulsion [59]. The shearing rheological properties of the protein emulsions under different pH values were analyzed by testing the apparent viscosity (Figure 12). With an increase in the shearing rate, the viscosity of the emulsions declined partially, and obvious shearing dilution was observed. This demonstrates that these emulsions are non-Newtonian fluids, and the flocculent structures of the emulsion droplets were destroyed in the shearing process. When the pH was close to the PI, the emulsions exhibited the highest viscosity and shearing dilution behavior due to the high flocculation of droplets, which is consistent with previous results [60]. For spherical oil droplets wrapped by proteins, the electrostatic repulsion among oil droplets decreases as the pH approaches the PI of the adsorbed protein, thus resulting in droplet clustering [61]. According to Stokes’ law, the degree of flocculation of an emulsion is positively related to the viscosity and degree of shearing dilution [62]. This is because the aggregate maintains some continuous phases in the structure, thus increasing the viscosity. Due to the external shearing effect, flocculation structural damage causes shearing dilution. Therefore, the viscosity of the Co emulsion was lower than that of PPI, CPI, and BL emulsions when the pH was higher than the PI (pH 7.0–11.0), indicating a low degree of flocculation of the droplets.

## 4. Conclusions

The comprehensive analysis shows that co-precipitation changes the subunit composition and hydrophobicity of mixed proteins, and the functional properties of Co and BL under deviating isoelectric points were synergistic. Additionally, the Co prepared by the ISP method exhibited better solubility, foamability, and emulsification than BL comprising a direct blend of isolated proteins, thereby indicating the advantages of the co-precipitation method. Further studies will be performed to investigate the functional properties of Co and protein modification under different temperatures and ion concentrations. This study provides a theoretical basis for further improvement of mixed protein processing methodology.

## Figures and Tables

**Table 1:** Relative band optical density (%) of BL and Co (non-reducing electrophoretogram).

Samples	Aggregates	MHC	Convicilin	Leg α + β	Vicilin	AC	Leg α	TM	Leg β	Others	Vicilin/ Leg α + β
BL	11.61	15.90	4.60	9.98	7.22	19.31	10.63	5.31	9.98	5.45	0.72
Co	19.62	15.27	0.11	6.55	13.36	16.46	8.06	5.36	4.63	10.57	2.04

**Table 2:** Average particle size of proteins at different pH values.

pH	Samples	*d*_4,3_ (μm)	*d*_3,2_ (μm)	*d′*_4,3_ (μm)	*d′*_3,2_ (μm)
3.0	PPI	10.66 ± 0.56 ^a^	6.75 ± 0.18 ^a^	3.68 ± 0.10 ^a^	2.63 ± 0.05 ^a^
	CPI	3.53 ± 0.06 ^c^	2.58 ± 0.10 ^b^	1.08 ± 0.10 ^c^	1.29 ± 0.07 ^c^
	BL	6.91 ± 0.06 ^b^	3.23 ± 1.56 ^b^	1.84 ± 0.07 ^b^	1.63 ± 0.06 ^b^
	Co	3.29 ± 0.27 ^c^	2.10 ± 0.07 ^b^	0.90 ± 0.01 ^c^	0.87 ± 0.01 ^d^
5.0	PPI	31.91 ± 3.23 ^a^	10.41 ± 1.01 ^a^	4.17 ± 0.13 ^b^	3.77 ± 0.19 ^a^
	CPI	9.85 ± 0.10 ^d^	4.28 ± 0.06 ^c^	2.69 ± 0.22 ^c^	2.01 ± 0.24 ^b^
	BL	17.12 ± 1.46 ^c^	8.21 ± 0.36 ^b^	4.16 ± 0.13 ^b^	3.96 ± 0.34 ^a^
	Co	22.79 ± 2.75 ^b^	9.33 ± 0.23 ^ab^	7.76 ± 0.18 ^a^	3.73 ± 0.10 ^a^
7.0	PPI	4.88 ± 0.19 ^a^	2.73 ± 0.05 ^a^	1.68 ± 0.03 ^a^	1.14 ± 0.07 ^a^
	CPI	2.57 ± 0.18 ^c^	1.56 ± 0.09 ^c^	0.77 ± 0.08 ^c^	0.63 ± 0.02 ^d^
	BL	3.96 ± 0.09 ^b^	2.46 ± 0.17 ^b^	1.45 ± 0.07 ^b^	1.00 ± 0.02 ^b^
	Co	1.17 ± 0.07 ^d^	0.84 ± 0.07 ^d^	0.60 ± 0.03 ^d^	0.85 ± 0.00 ^c^
9.0	PPI	3.20 ± 0.06 ^a^	2.89 ± 0.09 ^a^	1.28 ± 0.05 ^a^	1.16 ± 0.10 ^a^
	CPI	2.01 ± 0.06 ^b^	1.85 ± 0.11 ^b^	0.89 ± 0.02 ^c^	0.72 ± 0.06 ^b^
	BL	3.02 ± 0.14 ^a^	2.75 ± 0.10 ^a^	1.12 ± 0.01 ^b^	1.02 ± 0.05 ^a^
	Co	0.95 ± 0.01 ^d^	0.86 ± 0.00 ^c^	0.68 ± 0.01 ^d^	0.39 ± 0.01 ^d^
11.0	PPI	2.49 ± 0.05 ^a^	2.24 ± 0.19 ^a^	0.98 ± 0.03 ^a^	1.10 ± 0.07 ^a^
	CPI	0.93 ± 0.02 ^c^	0.87 ± 0.02 ^b^	0.47 ± 0.00 ^b^	0.35 ± 0.00 ^c^
	BL	1.15 ± 0.05 ^b^	1.09 ± 0.03 ^b^	0.57 ± 0.02 ^c^	0.47 ± 0.02 ^b^
	Co	0.55 ± 0.01 ^d^	0.53 ± 0.02 ^c^	0.33 ± 0.01 ^d^	0.20 ± 0.00 ^d^

Note: Each value represents the mean ± SD (*n* = 3). Different letters under the same pH indicate significant differences (*p* < 0.05).

**Table 3:** Interface protein adsorption and interface protein content of proteins at different pH values.

Samples	pH = 3.0		pH = 5		pH = 7		pH = 9		pH = 11	
	AP/%	Γ/ (mg/m^2^)	AP%	Γ/ (mg/m^2^)	AP%	Γ/ (mg/m^2^)	AP%	Γ/ (mg/m^2^)	AP%	Γ/ (mg/m^2^)
PPI	33.43 ± 1.64 ^b^	13.61 ± 0.07 ^a^	2.96 ± 0.72 ^b^	2.73 ± 0.01 ^d^	22.94 ± 3.66 ^c^	4.04 ± 0.10 ^c^	38.39 ± 2.69 ^b^	6.60 ± 0.03 ^a^	48.11 ± 1.57 ^b^	7.75 ± 0.02 ^a^
CPI	38.91 ± 4.37 ^ab^	8.13 ± 0.23 ^c^	11.42 ± 1.12 ^a^	3.05 ± 0.02 ^c^	33.35 ± 0.88 ^b^	3.11 ± 0.00 ^d^	36.21 ± 2.87 ^b^	3.95 ± 0.02 ^c^	54.94 ± 4.08 ^b^	2.91 ± 0.09 ^c^
BL	36.19 ± 2.01 ^b^	9.18 ± 0.06 ^b^	9.42 ± 2.26 ^a^	5.30 ± 0.04 ^a^	32.50 ± 3.64 ^b^	4.56 ± 0.12 ^b^	37.33 ± 1.45 ^b^	5.66 ± 0.02 ^b^	54.29 ± 3.70 ^b^	3.81 ± 0.05 ^b^
Co	44.15 ± 0.87 ^a^	5.65 ± 0.03 ^d^	8.63 ± 0.51 ^a^	6.57 ± 0.00 ^b^	48.91 ± 4.31 ^a^	6.06 ± 0.26 ^a^	51.71 ± 4.89 ^a^	3.13 ± 0.11 ^d^	65.61 ± 4.49 ^a^	2.03 ± 0.21 ^d^

Note: Each value represents the mean ± SD (*n* = 3). Different letters under the same pH value indicate significant differences (*p* < 0.05).

## Data Availability

Research data are not shared.
